# The challenges of managing antiretroviral treatment in children perinatally infected with HIV in Romania

**DOI:** 10.1186/1471-2334-14-S4-O4

**Published:** 2014-05-29

**Authors:** Alina Cibea, Mariana Mărdărescu, Cristina Petre, Ruxandra Neagu-Drăghicenoiu, Rodica Ungurianu, Sorin Petrea, Ana Maria Tudor, Delia Vlad, Carina Matei

**Affiliations:** 1 National Institute of Infectious Diseases “Prof. Dr. Matei Balş”, Bucharest, Romania

## 

In the past few years, the rate of HIV perinatally exposed children has increased as a consequence of the expanding number of infected women. These women belong, on the one hand, to Romania’s 1990s cohort, and on the other to the group of new sexually or intravenously (i.v.) drug infected women.

To report the characteristics of vertically HIV infected children in the last six years in Romania, to investigate temporal trends in their evolution with antiretroviral treatment and to characterize the causes of treatment failure in these children.

Children with perinatally acquired HIV infection were followed in a retrospective case series. The cohort included 43 children, evaluated in the National Institute of Infectious Diseases “Prof. Dr. Matei Balş”, born between 2008-2013, from 86 HIV infected children under 6 years, in active records in Romania. We assessed maternal characteristics, HIV vertical transmission prophylaxis, timing of diagnosis, immunological and virologic status, features of the evolution of these children with ART.

43 perinatally HIV infected children were evaluated in our clinic from 396 perinatally exposed children, with a rate of transmission of HIV infection of 10.8%. 16% of mothers belonged to the Romanian ’90s cohort and 84% were recently infected with HIV, sexually or through i.v. drug use. 41% of the subjects were diagnosed with HIV infection at birth. Their median entry CD4% was 23% and 49% had a CD4 >25%; median entry viral load was 7 log. 16 patients (37%) had undetectable viral load after six months of treatment. In 87.5% of them, the virologic suppression was achieved and maintained with one single regimen (2 NRTIs + 1 NNRTI or 2 NRTIs + 1 PI/r). 15 children (35%) had never achieved suppression of viral load to undetectable levels. 19 children (44%) faced special issues related to adherence to antiretroviral treatment.

The management of antiretroviral treatment in children with vertically acquired HIV infection is challenging, due to limited therapeutic options, lack of adherence, drug interactions and adverse events.

